# 6-Amino-2-methyl-8-phenyl-1,2,3,4-tetra­hydro­iso­quinoline-5,7-dicarbo­nitrile

**DOI:** 10.1107/S1600536813013342

**Published:** 2013-05-22

**Authors:** Dao-Cai Wang, Hang Song, Shun Yao

**Affiliations:** a Department of Pharmaceutical and Biological Engineering, College of Chemical Engineering, Sichuan University, Chengdu 610065, People’s Republic of China

## Abstract

In the title compound, C_18_H_16_N_4_, the dihedral angle between the benzene and phenyl rings is 61.40 (4)°. In the crystal, mol­ecules are linked by N—H⋯N(nitrile) hydrogen bonds, forming inversion dimers. The dimers are further linked by N—H⋯N(amine) hydrogen bonds, and both units are arranged almost perpendicular to each other [angle between dimer mean planes = 84.43 (12)°]. This arrangement is extended to form a ladder-like structure parallel to the *c* axis.

## Related literature

For background to natural products containing an iso­quinoline backbone, see: Marchand *et al.* (2006 ▶); Cho *et al.* (2007 ▶); Van Quaquebeke *et al.* (2007 ▶). For related structures, see: Rong *et al.* (2010 ▶); Balamurugan *et al.* (2011 ▶).
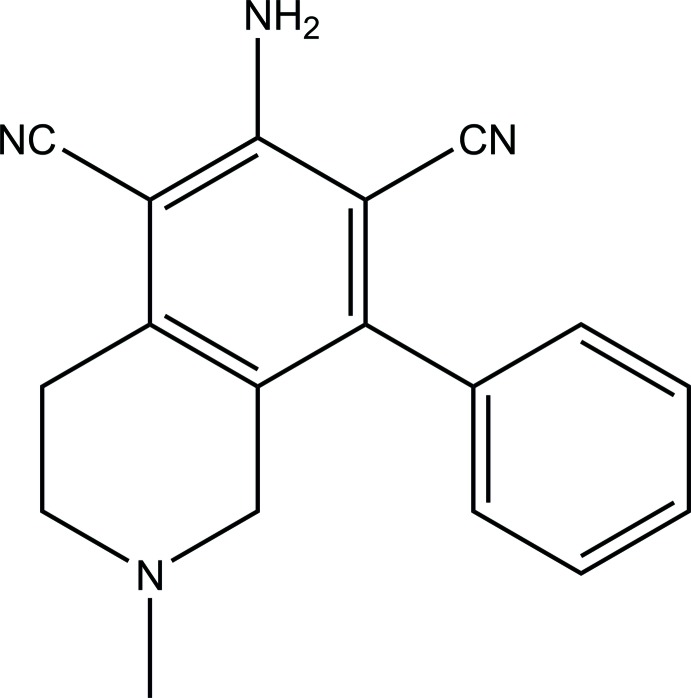


## Experimental

### 

#### Crystal data

C_18_H_16_N_4_
*M* *_r_* = 288.35
Monoclinic, 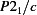

*a* = 17.5630 (5) Å
*b* = 6.25208 (19) Å
*c* = 13.7963 (4) Å
β = 93.209 (3)°
*V* = 1512.53 (8) Å^3^
*Z* = 4
Mo *K*α radiation
μ = 0.08 mm^−1^
*T* = 143 K
0.38 × 0.35 × 0.25 mm

#### Data collection

Agilent Xcalibur Eos diffractometer
Absorption correction: multi-scan (*CrysAlis PRO*; Oxford Diffraction, 2009 ▶) *T*_min_ = 0.960, *T*_max_ = 1.000
6799 measured reflections
3089 independent reflections
2414 reflections with *I* > 2σ(*I*)
*R*_int_ = 0.034

#### Refinement

*R*[*F* ^2^ > 2σ(*F* ^2^)] = 0.045
*wR*(*F* ^2^) = 0.112
*S* = 1.05
3089 reflections
206 parameters
4 restraints
H atoms treated by a mixture of independent and constrained refinement
Δρ_max_ = 0.19 e Å^−3^
Δρ_min_ = −0.22 e Å^−3^

### 

Data collection: *CrysAlis PRO* (Oxford Diffraction, 2009 ▶); cell refinement: *CrysAlis PRO*; data reduction: *CrysAlis PRO*; program(s) used to solve structure: *SHELXS97* (Sheldrick, 2008 ▶); program(s) used to refine structure: *SHELXL97* (Sheldrick, 2008 ▶); molecular graphics: *OLEX2* (Dolomanov *et al.*, 2009 ▶) and *Mercury* (Macrae *et al.*, 2008 ▶); software used to prepare material for publication: *OLEX2*.

## Supplementary Material

Crystal structure: contains datablock(s) I, global. DOI: 10.1107/S1600536813013342/bh2477sup1.cif

Structure factors: contains datablock(s) I. DOI: 10.1107/S1600536813013342/bh2477Isup2.hkl

Supplementary material file. DOI: 10.1107/S1600536813013342/bh2477Isup3.cml

Additional supplementary materials:  crystallographic information; 3D view; checkCIF report

## Figures and Tables

**Table 1:** Hydrogen-bond geometry (Å, °)

*D*—H⋯*A*	*D*—H	H⋯*A*	*D*⋯*A*	*D*—H⋯*A*
N4—H4*A*⋯N2^i^	0.89 (1)	2.21 (1)	3.052 (2)	157 (2)
N4—H4*B*⋯N1^ii^	0.87 (1)	2.21 (1)	3.0261 (19)	155 (2)

Symmetry codes: (i) 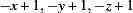; (ii) 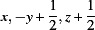.

**Fig. 1.**
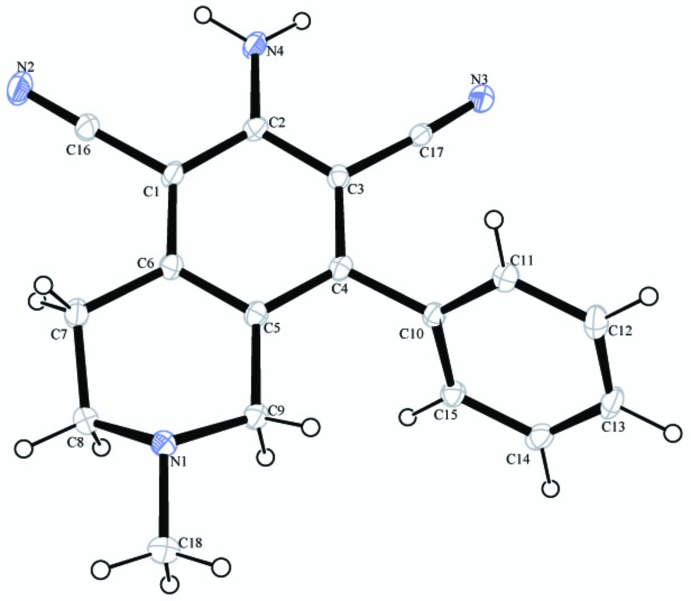
The molecular structure of the title compound, with displacement ellipsoids drawn at the 30% probability level.

**Fig. 2.**
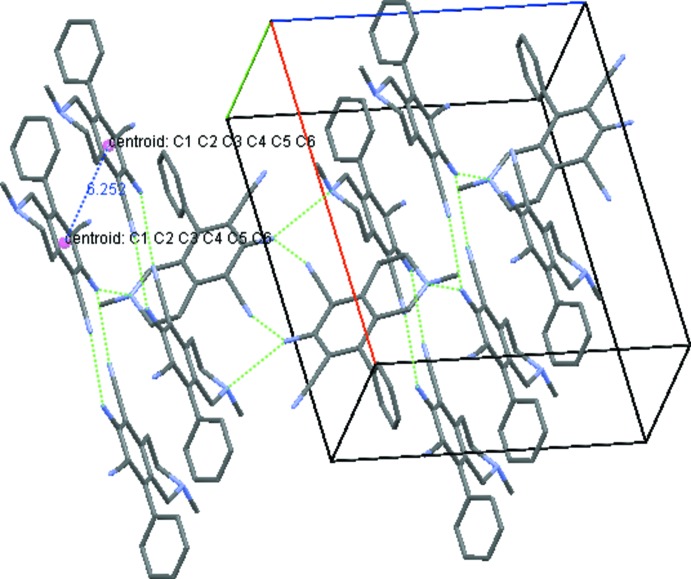
The crystal packing of the title compound. H atoms not involved in hydrogen bonding are omitted for clarity.

## supplementary crystallographic information

### Comment

Various natural products containing an isoquinoline backbone have received considerable attention over the past years, because of their different kinds of bioactivities (Marchand *et al.*, 2006; Cho *et al.*, 2007; Van Quaquebeke *et al.*, 2007). The development of efficient synthetic methods for isoquinoline and related derivatives is continuously attracting the attention of many chemists (Rong *et al.*, 2010; Balamurugan *et al.*, 2011). As a part of our current studies on the development of new routes in organic synthesis and the screen of anticancer drugs, in this article, we report the crystal structure of the title compound (Fig. 1).

The dihedral angle between the benzene and the phenyl rings is 61.40 (4)°. In the crystal (Fig. 2), two kinds of hydrogen bonds are formed, *i.e.*, *N*—*H*···*N*(nitrile group) and *N*—*H*···*N*(amine) hydrogen bonds, connecting molecules in different directions. The *N*—*H*···*N*(nitrile group) hydrogen bonds are involved in the formation of centrosymmetric dimers, and this basic unit is linked by *N*—*H*···*N*(amine) hydrogen bonds to four other symmetry-related units, which are parallel to each other. Non-parallel units are arranged almost perpendicular to each other [angle between unit mean planes: 84.43 (12)°] forming a ladder structure, that can extend into infinite sheets through the vertical direction of the step cross section. On the step cross section, the distance between the centers of two units is 6.252 (13) Å.

### Experimental

Benzaldehyde (1 mmol) was added to a stirred solution of 1-methyl-4-piperidone (1 mmol) and tetrahydro pyrrole (1.2 mmol) in dichloromethane. The resulting mixture was refluxed for 4 h to obtain a α,β-unsaturated ketone as an intermediate. Subsequently, this α,β-unsaturated ketone (1 mmol) was reacted with malononitrile (2 mmol) under strong basic conditions (Rong *et al.*, 2009), monitoring the reaction progress by TLC. After the completion of the reaction, the solvent was evaporated under reduce pressure and the crude product was purified by silica gel column chromatography. Crystals suitable for X-ray analysis were obtained by slow evaporation of a solution in methanol and dichloromethane (4:1).

### Refinement

Carbon-bound H-atoms were placed in calculated positions [C—H = 0.95–0.99 Å, *U*_iso_(H) = 1.2–1.5*U*_eq_(parent atom)] and were allowed to ride on their parent atoms. The amino-H atoms H4A and H4B were located in a difference map, and subsequently refined freely, with *U*_iso_(H) = 1.2*U*_eq_(N4).

### Crystal data

**Table:**

C_18_H_16_N_4_	*F*(000) = 608
*M**_r_* = 288.35	*D*_x_ = 1.266 Mg m^−^^3^
Monoclinic, *P*2_1_/*c*	Mo *K*α radiation, λ = 0.7107 Å
*a* = 17.5630 (5) Å	Cell parameters from 2345 reflections
*b* = 6.25208 (19) Å	θ = 3.0–29.0°
*c* = 13.7963 (4) Å	µ = 0.08 mm^−^^1^
β = 93.209 (3)°	*T* = 143 K
*V* = 1512.53 (8) Å^3^	Block, colourless
*Z* = 4	0.38 × 0.35 × 0.25 mm

### Data collection

**Table:**

Agilent Xcalibur Eos diffractometer	3089 independent reflections
Radiation source: Enhance (Mo) X-ray Source	2414 reflections with *I* > 2σ(*I*)
Graphite monochromator	*R*_int_ = 0.034
Detector resolution: 16.0874 pixels mm^-1^	θ_max_ = 26.4°, θ_min_ = 3.0°
ω scans	*h* = −21→21
Absorption correction: multi-scan (*CrysAlis PRO*; Oxford Diffraction, 2009)	*k* = −7→7
*T*_min_ = 0.960, *T*_max_ = 1.000	*l* = −16→17
6799 measured reflections	

### Refinement

**Table:**

Refinement on *F*^2^	Primary atom site location: structure-invariant direct methods
Least-squares matrix: full	Secondary atom site location: difference Fourier map
*R*[*F*^2^ > 2σ(*F*^2^)] = 0.045	Hydrogen site location: inferred from neighbouring sites
*wR*(*F*^2^) = 0.112	H atoms treated by a mixture of independent and constrained refinement
*S* = 1.05	*w* = 1/[σ^2^(*F*_o_^2^) + (0.0403*P*)^2^ + 0.3434*P*] where *P* = (*F*_o_^2^ + 2*F*_c_^2^)/3
3089 reflections	(Δ/σ)_max_ < 0.001
206 parameters	Δρ_max_ = 0.19 e Å^−^^3^
4 restraints	Δρ_min_ = −0.22 e Å^−^^3^
0 constraints	

### Fractional atomic coordinates and isotropic or equivalent isotropic displacement parameters (Å^2^)

**Table:**

	*x*	*y*	*z*	*U*_iso_*/*U*_eq_	
N1	0.29081 (7)	−0.2394 (2)	0.20203 (9)	0.0244 (3)	
N2	0.50947 (9)	0.2855 (3)	0.43619 (13)	0.0498 (5)	
N3	0.14961 (8)	0.4536 (2)	0.57681 (10)	0.0308 (3)	
N4	0.34160 (8)	0.4642 (2)	0.53715 (11)	0.0323 (4)	
H4A	0.3908 (8)	0.501 (3)	0.5410 (12)	0.039*	
H4B	0.3136 (9)	0.524 (3)	0.5798 (11)	0.039*	
C1	0.36603 (8)	0.1934 (2)	0.41825 (11)	0.0234 (3)	
C2	0.31623 (9)	0.3112 (2)	0.47415 (11)	0.0223 (3)	
C3	0.23791 (8)	0.2589 (2)	0.46164 (10)	0.0201 (3)	
C4	0.21118 (8)	0.0990 (2)	0.39679 (10)	0.0199 (3)	
C5	0.26291 (8)	−0.0177 (2)	0.34345 (10)	0.0207 (3)	
C6	0.34051 (8)	0.0294 (2)	0.35473 (10)	0.0221 (3)	
C7	0.39820 (9)	−0.0919 (3)	0.29916 (12)	0.0288 (4)	
H7A	0.4417	−0.1330	0.3440	0.035*	
H7B	0.4179	0.0024	0.2486	0.035*	
C8	0.36428 (9)	−0.2911 (3)	0.25165 (12)	0.0274 (4)	
H8A	0.3994	−0.3483	0.2043	0.033*	
H8B	0.3572	−0.4021	0.3015	0.033*	
C9	0.23529 (9)	−0.1913 (2)	0.27405 (11)	0.0238 (4)	
H9A	0.2251	−0.3227	0.3113	0.029*	
H9B	0.1868	−0.1457	0.2403	0.029*	
C10	0.12744 (8)	0.0567 (2)	0.38652 (10)	0.0205 (3)	
C11	0.07739 (9)	0.2175 (3)	0.35361 (11)	0.0251 (4)	
H11	0.0967	0.3541	0.3374	0.030*	
C12	−0.00049 (9)	0.1790 (3)	0.34448 (11)	0.0300 (4)	
H12	−0.0343	0.2889	0.3217	0.036*	
C13	−0.02895 (9)	−0.0187 (3)	0.36837 (11)	0.0306 (4)	
H13	−0.0823	−0.0446	0.3623	0.037*	
C14	0.02015 (9)	−0.1791 (3)	0.40108 (11)	0.0292 (4)	
H14	0.0005	−0.3152	0.4174	0.035*	
C15	0.09809 (9)	−0.1418 (2)	0.41021 (11)	0.0243 (4)	
H15	0.1316	−0.2526	0.4328	0.029*	
C16	0.44596 (9)	0.2426 (3)	0.42808 (12)	0.0311 (4)	
C17	0.18663 (8)	0.3655 (2)	0.52358 (11)	0.0221 (3)	
C18	0.26348 (10)	−0.4182 (3)	0.14143 (12)	0.0344 (4)	
H18A	0.2595	−0.5464	0.1817	0.052*	
H18B	0.2993	−0.4447	0.0909	0.052*	
H18C	0.2132	−0.3833	0.1111	0.052*	

### Atomic displacement parameters (Å^2^)

**Table:**

	*U*^11^	*U*^22^	*U*^33^	*U*^12^	*U*^13^	*U*^23^
N1	0.0234 (7)	0.0257 (7)	0.0243 (7)	−0.0017 (6)	0.0042 (5)	−0.0057 (5)
N2	0.0229 (8)	0.0629 (11)	0.0644 (11)	−0.0121 (8)	0.0114 (7)	−0.0318 (9)
N3	0.0234 (7)	0.0328 (8)	0.0363 (8)	−0.0017 (6)	0.0042 (6)	−0.0068 (6)
N4	0.0208 (7)	0.0385 (8)	0.0383 (8)	−0.0093 (7)	0.0084 (6)	−0.0166 (7)
C1	0.0173 (8)	0.0276 (8)	0.0255 (8)	−0.0043 (6)	0.0033 (6)	−0.0005 (7)
C2	0.0215 (8)	0.0235 (8)	0.0222 (8)	−0.0039 (6)	0.0021 (6)	0.0006 (6)
C3	0.0191 (8)	0.0206 (8)	0.0207 (7)	−0.0003 (6)	0.0029 (6)	0.0023 (6)
C4	0.0185 (7)	0.0207 (8)	0.0204 (7)	−0.0016 (6)	0.0008 (6)	0.0056 (6)
C5	0.0198 (8)	0.0219 (8)	0.0205 (7)	−0.0014 (6)	0.0018 (6)	0.0025 (6)
C6	0.0202 (8)	0.0246 (8)	0.0217 (7)	−0.0009 (6)	0.0033 (6)	0.0008 (6)
C7	0.0206 (8)	0.0365 (9)	0.0298 (9)	−0.0008 (7)	0.0044 (6)	−0.0061 (7)
C8	0.0242 (9)	0.0277 (9)	0.0306 (9)	0.0022 (7)	0.0046 (7)	−0.0037 (7)
C9	0.0214 (8)	0.0255 (8)	0.0248 (8)	−0.0027 (6)	0.0034 (6)	−0.0015 (6)
C10	0.0186 (8)	0.0250 (8)	0.0180 (7)	−0.0007 (6)	0.0021 (6)	−0.0029 (6)
C11	0.0228 (8)	0.0285 (9)	0.0239 (8)	−0.0003 (7)	0.0009 (6)	0.0007 (7)
C12	0.0203 (8)	0.0426 (10)	0.0271 (9)	0.0060 (7)	−0.0002 (6)	−0.0023 (7)
C13	0.0160 (8)	0.0494 (11)	0.0265 (8)	−0.0053 (7)	0.0036 (6)	−0.0102 (8)
C14	0.0267 (9)	0.0336 (9)	0.0281 (9)	−0.0095 (7)	0.0080 (7)	−0.0065 (7)
C15	0.0228 (8)	0.0256 (8)	0.0246 (8)	−0.0022 (7)	0.0039 (6)	−0.0009 (7)
C16	0.0234 (9)	0.0353 (9)	0.0353 (9)	−0.0050 (7)	0.0071 (7)	−0.0142 (8)
C17	0.0189 (8)	0.0210 (8)	0.0260 (8)	−0.0042 (6)	−0.0011 (6)	0.0005 (7)
C18	0.0331 (10)	0.0334 (10)	0.0369 (10)	−0.0041 (8)	0.0037 (7)	−0.0119 (8)

### Geometric parameters (Å, º)

**Table:**

N1—C8	1.463 (2)	C7—H7B	0.9900
N1—C9	1.4618 (19)	C7—C8	1.514 (2)
N1—C18	1.4607 (19)	C8—H8A	0.9900
N2—C16	1.147 (2)	C8—H8B	0.9900
N3—C17	1.1483 (19)	C9—H9A	0.9900
N4—H4A	0.893 (12)	C9—H9B	0.9900
N4—H4B	0.874 (12)	C10—C11	1.394 (2)
N4—C2	1.3510 (19)	C10—C15	1.390 (2)
C1—C2	1.406 (2)	C11—H11	0.9500
C1—C6	1.406 (2)	C11—C12	1.388 (2)
C1—C16	1.436 (2)	C12—H12	0.9500
C2—C3	1.415 (2)	C12—C13	1.380 (2)
C3—C4	1.405 (2)	C13—H13	0.9500
C3—C17	1.439 (2)	C13—C14	1.381 (2)
C4—C5	1.405 (2)	C14—H14	0.9500
C4—C10	1.4931 (19)	C14—C15	1.387 (2)
C5—C6	1.394 (2)	C15—H15	0.9500
C5—C9	1.510 (2)	C18—H18A	0.9800
C6—C7	1.509 (2)	C18—H18B	0.9800
C7—H7A	0.9900	C18—H18C	0.9800
			
C9—N1—C8	109.38 (12)	C7—C8—H8B	109.7
C18—N1—C8	110.60 (12)	H8A—C8—H8B	108.2
C18—N1—C9	109.63 (12)	N1—C9—C5	112.08 (12)
H4A—N4—H4B	115.4 (16)	N1—C9—H9A	109.2
C2—N4—H4A	120.3 (11)	N1—C9—H9B	109.2
C2—N4—H4B	124.1 (11)	C5—C9—H9A	109.2
C2—C1—C16	118.09 (14)	C5—C9—H9B	109.2
C6—C1—C2	122.51 (14)	H9A—C9—H9B	107.9
C6—C1—C16	119.40 (14)	C11—C10—C4	120.20 (13)
N4—C2—C1	122.05 (14)	C15—C10—C4	120.79 (13)
N4—C2—C3	121.70 (14)	C15—C10—C11	119.01 (14)
C1—C2—C3	116.25 (13)	C10—C11—H11	119.9
C2—C3—C17	117.18 (13)	C12—C11—C10	120.30 (15)
C4—C3—C2	122.01 (13)	C12—C11—H11	119.9
C4—C3—C17	120.65 (13)	C11—C12—H12	119.9
C3—C4—C5	120.01 (13)	C13—C12—C11	120.15 (15)
C3—C4—C10	118.59 (13)	C13—C12—H12	119.9
C5—C4—C10	121.39 (13)	C12—C13—H13	120.0
C4—C5—C9	120.75 (13)	C12—C13—C14	120.00 (15)
C6—C5—C4	119.25 (13)	C14—C13—H13	120.0
C6—C5—C9	120.00 (13)	C13—C14—H14	119.9
C1—C6—C7	118.93 (13)	C13—C14—C15	120.17 (15)
C5—C6—C1	119.93 (13)	C15—C14—H14	119.9
C5—C6—C7	121.13 (13)	C10—C15—H15	119.8
C6—C7—H7A	109.2	C14—C15—C10	120.37 (15)
C6—C7—H7B	109.2	C14—C15—H15	119.8
C6—C7—C8	111.98 (13)	N2—C16—C1	178.82 (19)
H7A—C7—H7B	107.9	N3—C17—C3	175.71 (15)
C8—C7—H7A	109.2	N1—C18—H18A	109.5
C8—C7—H7B	109.2	N1—C18—H18B	109.5
N1—C8—C7	109.66 (13)	N1—C18—H18C	109.5
N1—C8—H8A	109.7	H18A—C18—H18B	109.5
N1—C8—H8B	109.7	H18A—C18—H18C	109.5
C7—C8—H8A	109.7	H18B—C18—H18C	109.5
			
N4—C2—C3—C4	−179.53 (14)	C6—C1—C2—C3	−1.3 (2)
N4—C2—C3—C17	−4.0 (2)	C6—C1—C16—N2	147 (9)
C1—C2—C3—C4	−0.3 (2)	C6—C5—C9—N1	−20.08 (19)
C1—C2—C3—C17	175.24 (13)	C6—C7—C8—N1	46.68 (18)
C1—C6—C7—C8	167.68 (14)	C8—N1—C9—C5	54.58 (16)
C2—C1—C6—C5	1.8 (2)	C9—N1—C8—C7	−69.32 (16)
C2—C1—C6—C7	−178.69 (14)	C9—C5—C6—C1	179.00 (13)
C2—C1—C16—N2	−34 (9)	C9—C5—C6—C7	−0.5 (2)
C2—C3—C4—C5	1.3 (2)	C10—C4—C5—C6	179.26 (13)
C2—C3—C4—C10	−178.81 (13)	C10—C4—C5—C9	−0.3 (2)
C2—C3—C17—N3	−19 (2)	C10—C11—C12—C13	0.3 (2)
C3—C4—C5—C6	−0.9 (2)	C11—C10—C15—C14	0.1 (2)
C3—C4—C5—C9	179.50 (13)	C11—C12—C13—C14	−0.3 (2)
C3—C4—C10—C11	61.30 (18)	C12—C13—C14—C15	0.1 (2)
C3—C4—C10—C15	−118.16 (16)	C13—C14—C15—C10	0.0 (2)
C4—C3—C17—N3	157 (2)	C15—C10—C11—C12	−0.2 (2)
C4—C5—C6—C1	−0.6 (2)	C16—C1—C2—N4	−1.3 (2)
C4—C5—C6—C7	179.86 (13)	C16—C1—C2—C3	179.48 (14)
C4—C5—C9—N1	159.53 (13)	C16—C1—C6—C5	−179.03 (14)
C4—C10—C11—C12	−179.67 (14)	C16—C1—C6—C7	0.5 (2)
C4—C10—C15—C14	179.53 (13)	C17—C3—C4—C5	−173.99 (13)
C5—C4—C10—C11	−118.85 (16)	C17—C3—C4—C10	5.9 (2)
C5—C4—C10—C15	61.68 (19)	C18—N1—C8—C7	169.84 (13)
C5—C6—C7—C8	−12.8 (2)	C18—N1—C9—C5	176.02 (13)
C6—C1—C2—N4	177.97 (15)		

### Hydrogen-bond geometry (Å, º)

**Table:**

*D*—H···*A*	*D*—H	H···*A*	*D*···*A*	*D*—H···*A*
N4—H4*A*···N2^i^	0.89 (1)	2.21 (1)	3.052 (2)	157 (2)
N4—H4*B*···N1^ii^	0.87 (1)	2.21 (1)	3.0261 (19)	155 (2)

Symmetry codes: (i) −*x*+1, −*y*+1, −*z*+1; (ii) *x*, −*y*+1/2, *z*+1/2.

## References

[bb1] Balamurugan, K., Jeyachandran, V., Perumal, S. & Menéndez, J. C. (2011). *Tetrahedron*, **67**, 1432–1437.

[bb2] Cho, W.-J., Le, Q. M., Van, H. T. M., Lee, K. Y., Kang, B. Y., Lee, E.-S., Lee, S. K. & Kwon, Y. (2007). *Bioorg. Med. Chem. Lett.* **17**, 3531–3534. 10.1016/j.bmcl.2007.04.064 17498951

[bb3] Dolomanov, O. V., Bourhis, L. J., Gildea, R. J., Howard, J. A. K. & Puschmann, H. (2009). *J. Appl. Cryst.* **42**, 339–341.

[bb4] Macrae, C. F., Bruno, I. J., Chisholm, J. A., Edgington, P. R., McCabe, P., Pidcock, E., Rodriguez-Monge, L., Taylor, R., van de Streek, J. & Wood, P. A. (2008). *J. Appl. Cryst.* **41**, 466–470.

[bb5] Marchand, C., Antony, S., Kohn, K. W., Cushman, M., Ioanoviciu, A., Staker, B. L., Burgin, A. B., Stewart, L. & Pommier, Y. (2006). *Mol. Cancer Ther.* **5**, 287–295. 10.1158/1535-7163.MCT-05-0456 PMC2860177 16505102

[bb6] Oxford Diffraction (2009). *CrysAlis PRO* Oxford Diffraction, Yarnton, England.

[bb7] Rong, L. C., Gao, L. J., Han, H. X., Jiang, H., Dai, Y. S. & Tu, S. J. (2010). *Synth. Commun.* **40**, 289–294.

[bb8] Sheldrick, G. M. (2008). *Acta Cryst.* A**64**, 112–122. 10.1107/S0108767307043930 18156677

[bb9] Van Quaquebeke, E., Mahieu, T., Dumont, P., Dewelle, J., Ribaucour, F., Simon, G., Sauvage, S., Gaussin, J.-F., Tuti, J., El Yazidi, M., Van Vynckt, F., Mijatovic, T., Lefranc, F., Darro, F. & Kiss, R. (2007). *J. Med. Chem.* **50**, 4122–4134. 10.1021/jm070315q 17658777

